# Proteomics and Its Applications in Cancers

**DOI:** 10.3390/ijms24054457

**Published:** 2023-02-24

**Authors:** Stanislav Naryzhny

**Affiliations:** 1 Institute of Biomedical Chemistry, Pogodinskaya, 10, 119121 Moscow, Russia; snaryzhny@mail.ru; 2 Petersburg Institute of Nuclear Physics (PNPI) of National Research Center “Kurchatov Institute”, 188300 Gatchina, Russia

Cancer is a system malignant transformation that covers a wide group of diseases and can affect any organ of the human body. Cancer is one of the leading causes of mortality worldwide. The specific feature of cancer is a cellular reorganization that causes rapid, uncontrolled proliferation. Just a single abnormal cell can produce a tumor [1]. The tumor may penetrate surrounding tissues and migrate to other organs, i.e., metastasize. Proteomics can generate detailed information on the situation with the human proteome during this malignant transformation. For example, it unravels key information, which can contribute to the search for clinically applicable biomarkers and therapeutic targets. Proteomics can give answers not only about the number and abundance of proteins but also about their involvement in metabolic pathways, interactions, post-translational modifications (PTMs), and synthesis and degradation. Many other features of proteomics can be used in the study of cancer as well. Together with other omics studies, proteomics is providing extensive data related to molecular mechanisms of cancer. Based on extensive data generated by proteomics methods, cancer databases have been organized [2]. Our Special Issue (SI) was conceived to facilitate the publication of the latest papers in the field of cancer proteomics. A collection of seven articles presented in this SI offers very different examples of the most recent proteomics advances in the study of cancer.

The review paper of Arias-Hidalgo et al. is about single-cell proteomics (SCP) analysis [3]. SCP is used in many biomedical aspects, including cancer immunotherapy or biomarkers. Here, to study proteins as accurately as possible, advanced approaches in SCP are needed. Sample preparation is critical, as it influences the reliability of liquid chromatography–mass spectrometry (LC-MS) analysis. The review describes several methods that facilitate the processing of samples at the nanoscale and allows the quantitative determination of more than a thousand proteins in individual cells.

The interesting proteomics application in cancer vaccine development was presented by Lokhov et al. [4]. Recently, a new proteomics platform that enables the production of antigen compositions called “antigenic essences” has been developed. This approach has been used to produce cancer vaccines. Here, to guarantee similarity, the antigenic essence is accurately controlled by peptide mass spectrometry. Therefore, the proteomics platform has a high potential to improve the currently used cancer vaccines. In their paper, Lokhov et al. draw the attention of the science community to this innovative platform. Together with a summary of the approach and a list of cancer vaccines appropriate to be upgraded, this paper provides the main issues of the vaccine update using antigenic essences. 

The article by Cheng Ma et al. concerns anti-cancer medication [5]. Complex changes in glycosylation and protein levels underlie the various effects of the antitumor drug 2-deoxy-D-glucose (2DG) on cancer cells. 2DG inhibits the growth and survival of cancer cells by interfering with the ATP produced during D-glucose metabolism. In addition, 2DG inhibits protein glycosylation in vivo by competing with D-mannose, leading to endoplasmic reticulum (ER) stress responses in cancer cells. Using the HT29 colorectal cancer cell line as a model system, Cheng Ma et al. characterized 2DG-induced changes in N-glycosylation. 2DG treatment also functionally disrupted the global cellular proteome, as evidenced by significant activation of proteins involved in protein folding, endoplasmic reticulum, mitochondrial function, cellular respiration, oxidative phosphorylation, and translation. Taken together, these results reveal the complex changes in the cancer proteome that underlie the various effects of 2DG on cancer cells and may provide important clues to the development of therapeutics targeting protein glycosylation. This new information may provide clues to therapeutic developments targeting protein glycosylation.

There are a couple of papers in the SI that are in a rather different proteomics area—a functional role of a single protein in connection to the whole proteome [6,7]. Michalak et al. investigate the role of galectin-4 (Gal4) in colorectal cancer (CRC) [6]. To explore its function in CRC, Michalak et al. have established a CRC cell line with the possibility of Gal4 expression regulation. Using this model in combination with proteomics and phosphoproteomics analyses, they have screened the intracellular changes induced by Gal4 expression. They have identified and quantified 3083 proteins and 2071 phosphosites. They found that the top candidates that were modulated by Gal4 are PURB, MAPKAPK3, BTF3 and BCAR1, while the prime candidates with altered phosphorylation included ZBTB7A, FOXK1, PURB and CK2beta. The data presented by Torres et al. show that haptoglobin (HP) can be considered a new member of the inducible damage-associated molecular patterns (DAMPs) with an important role in vitro dendritic cells (DC) activation for cancer immunotherapy [7]. DAMPs are conserved endogenous factors that can act as “danger signals” and induce an inflammatory response [8]. The data of Torres et al. support the idea that HP induces a specific proteomics profile and a mature-associated phenotype on generated primary human monocyte-derived DC. It allows us to consider HP as a DAMP molecule. This information could be used in developing new ex vivo manipulation of DC for therapeutic purposes, not only treatment of the cancer, but also autoimmune and neurodegenerative diseases. 

The review paper of Tutanov and Tamkovich is about the combining effects of circulating DNA and proteins [9]. Circulating DNA is already used as a valuable tool in translational medicine. However, the association of this DNA with different proteins was not analyzed so far, despite considerable evidence that this association might impact circulation and the biological role of DNA. Tutanov and Tamkovich have collected and analyzed the current information about circulating DNA’s origins and forms of circulation, known biological effects, and the clinical potential of circulating tumor DNA–protein complexes.

Finally, the paper “Construction of 2DE Patterns of Plasma Proteins: Aspect of Potential Tumor Markers” [10] is focused on the classical aspects of cancer proteomics—a search for clinically applicable biomarkers and new therapeutic targets. The use of tumor markers aids in cancer treatment prognosis and recurrence. There is also hope that they might be useful in screening tests for the early detection of cancer. Here, the finding of ideal tumor markers, which should be sensitive, specific, and reliable, is an acute issue. Human plasma analysis is one of the most popular assays in clinics. Plasma proteomics can be useful to follow any changes in the human body, including cancer. Here, the availability of reliable control of proteoform patterns is a very important issue.

Altogether, this SI presents two very interesting reviews and five research papers covering a wide scientific area, in which cancer proteomics is involved. As a guest editor, I would like to thank all contributors, peer reviewers, editors, and MDPI´s publishing team for their efforts. I also hope that this SI will be of interest to the scientific community.
